# Spin-dependent thermoelectric effects in Fe-C_6_ doped monolayer MoS_2_

**DOI:** 10.1038/s41598-017-00599-6

**Published:** 2017-03-29

**Authors:** Lin Zhu, Fei Zou, Guoying Gao, Kailun Yao

**Affiliations:** School of Physics and Wuhan National High Magnetic Field Center, Huazhong University of Science and Technology, Wuhan, 430074 China

## Abstract

By using the non-equilibrium Green’s function with density functional theory, we have studied the thermal spin transport properties of Fe-C_6_ cluster doped monolayer MoS_2_. The results show that the device has a perfect Seebeck effect under temperature difference without gate voltage or bias voltage. Moreover, we also find the thermal colossal magnetoresistance effect, which is as high as 10^7^%. The competition between spin up electrons and spin down holes of the parallel spin configuration leads to peculiar behavior of colossal magnetoresistance and thermo-current, which is essential for the design of thermal transistors. These results are useful in future MoS_2_-based multifunctional spin caloritronic devices.

## Introduction

Spin caloritronics aims to explore the coupling and application of spins and charges with heat currents in materials^1^, which has potential applications in future technologies, such as green energy and information science. Encouraged by experimentally inversing spin Hall effect (ISHE)^2–4^ based physical measurements, spin caloritronics should treat an invigorated intersection of spintronics and thermoelectronics. Especially, unlike the spin polarized current relying on the bias voltage in spintronics, the thermally induced spin current is just generated from a temperature gradient, instead of electrical bias. In the field, spin Seebeck effect (SSE) is an inspiring discovery in ferromagnetic metal and semiconductor *et al*.^5–9^, which can achieve a conversion between heat and electricity, then provides an effective way and a new direction to explore and utilize new materials and green energy. Moreover, if spin-up and spin-down thermo-currents with nearly equal magnitudes flow in opposite directions, the perfect SSE occurs. Perfect SSE devices allow us to obtain the net spin current, decreasing dissipation heat caused by the total charge current. Therefore, we can design low-power consumption devices based on spin caloritronics. However, a huge challenge to realize the perfect SSE is to search the good spin caloritronic material.

The monolayer MoS_2_ and the related two-dimensional materials exhibit superior caloritronic performance^10–12^, they have attracted much attention^13–15^. Different from graphene and silicene, monolayer MoS_2_ has a direct energy gap of 1.8 eV^16, 17^. When transition metal atom is doped, the dopant 3d states present in the energy gap, then the magnetism of the doped system can be controlled by regulating 3d states of transition metal. Recent experiments successfully doped transition metals in monolayer MoS_2_^18^. There are also some theoretical studies on transition metals or atomic clusters doped monolayer MoS_2_^19–22^. Cheng *et al*.^19^ used the first-principles method to predict that the two-dimensional dilute magnetic semiconductors (DMSs) were easy to achieve by substitution of Mo with Mn, Fe, Co or Zn atom in monolayer MoS_2_. Feng *et al*.^21^ considered that the six S atoms in the vicinity of Mo could be replaced by other non-metallic elements (such as C, O, etc.), and they doped monolayer MoS_2_ with Fe-C_6_ and Fe-O_6_ clusters acquiring two DMSs. The devices based on MoS_2_ had also been designed^23–31^.

Here, we proposed a Fe-C_6_ cluster doped monolayer MoS_2_ system with an intriguing thermally induced colossal magneto-resistance (CMR) without gate voltage, finding the negative thermal magnetoresistance. Meanwhile, the perfect SSE and nonlinear response behavior of charge current were also observed.

## Model and Methods

**Figure 1:**
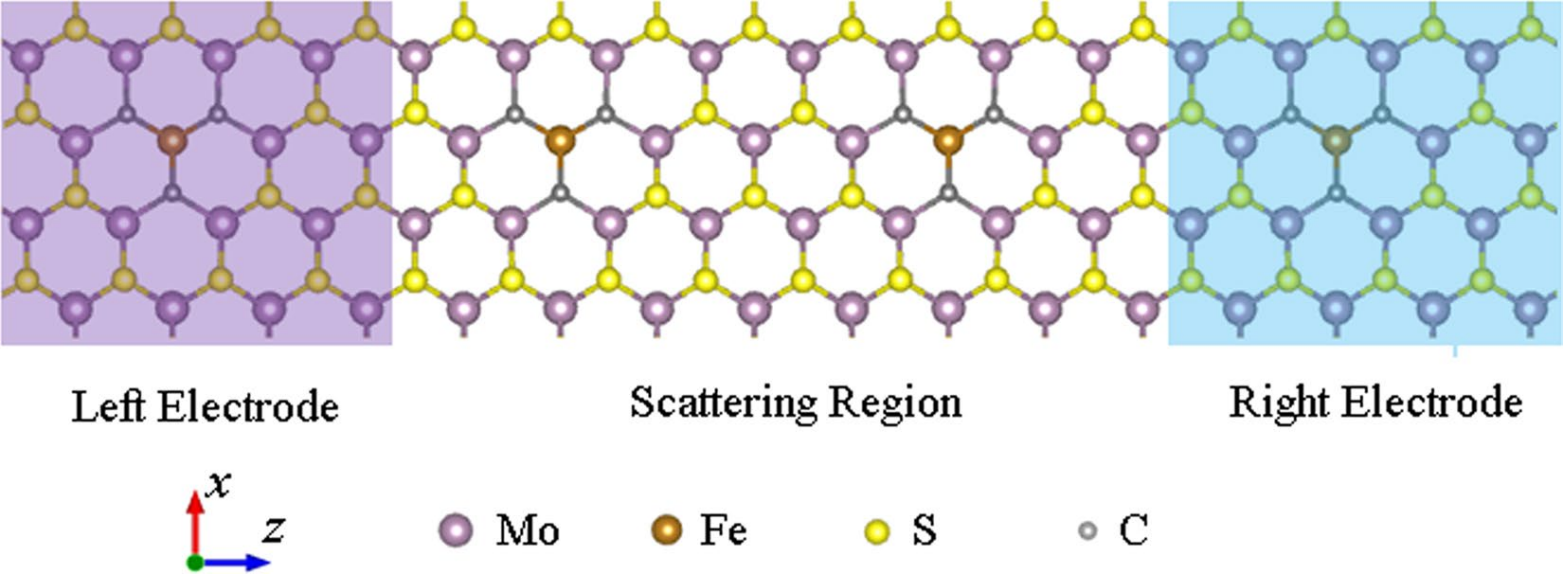
Schematic illustration of two-probe system. Schematic illustration of Fe-C_6_ cluster doped monolayer MoS_2_ two-probe system.

The model device displayed in Fig. 1 was divided into three parts: the left and right electrodes, and the scattering region. A 4 × 4 × 1 supercell model by replacing Mo-S_6_ with Fe-C_6_ was built. For convenience, we label the device with the symbol Fe-C_6_.

The transport calculations were performed within the framework of the QuantumWise ATK package^32–34^. The Perdew-Burke-Ernzernhof (PBE)^35^ spin-polarized generalized gradient approximation (SGGA) was used for the exchange-correlation potential, and the valence electronic orbitals were expanded in double-polarized basis set. The thermally induced current was given by^36^

1 $${{I}}^{\sigma }=\frac{e}{h}{\int }_{-\infty }^{+\infty }\{{{T}}^{\sigma }({E})[{{f}}_{L}({E},{{T}}_{L})-{{f}}_{R}({E},{{T}}_{R})]\}d{E}$$

here σ (=↑, ↓) denotes the spin index, and *μ*_*L*/*R*_ is the electrochemical potential for source/drain. As we just considered the temperature difference without gate voltage or bias voltage, then *μ*_*L*_ = *μ*_*R*_ = *E*_F_ (Fermi level) was set to zero. ${T}^{\sigma }(E)=Tr{({{\rm{\Gamma }}}_{L}{G}^{R}{{\rm{\Gamma }}}_{R}{G}^{A})}^{\sigma }$ is spin-resolved transmission function.

The system was relaxed sufficiently till the maximum force dropped below a threshold value of 0.01 eV/Å. Experimentally, the magnetization of the left and right electrodes can be aligned in parallel (P) or antiparallel (AP) spin configuration by a sufficiently strong external magnetic field. Therefore, the P and AP spin configurations were both considered. The total energy of AP spin configuration was 0.88 meV less than that of the P spin configuration per unit cell.

## Results and Discussion

**Figure 2:**
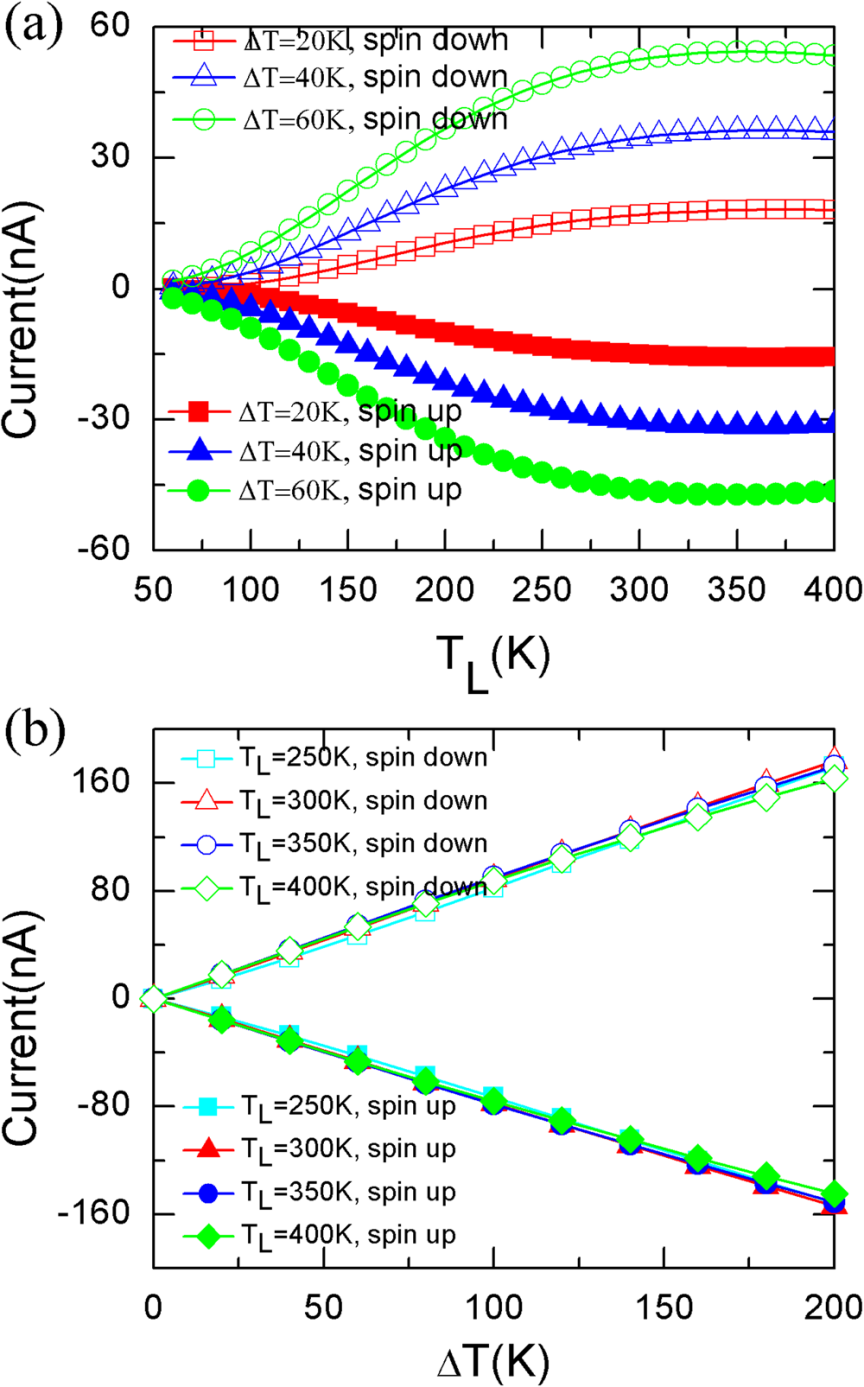
The spin-resolved thermal current. The spin-dependent thermal currents *versus T*_*L*_ for different *∆T* (Δ*T* = *T*_*R*_ − *T*_*L*_) (**a**) and *versus ∆T* for different *T*_*L*_ (**b**) for P spin configuration.

Figure 2 displays the thermally induced currents of Fe-C_6_ device *versus T*_*L*_ with different *∆T* (Δ*T* = *T*_*R*_ − *T*_*L*_), and these *versus ∆T* at different *T*_*L*_ for P spin configuration. The trend of curve is almost identical for spin-up or spin-down state, and there are no temperature threshold. The spin-polarized currents increase rapidly in low *T*_*L*_ region and slowly in high *T*_*L*_ region for all *∆T* (Fig. 2(a)), but the currents almost linearly increase in the whole range of *∆T* for all *T*_*L*_ (Fig. 2(b)). The different sign of spin-up and spin-down currents implies SSE.

**Figure 3:**
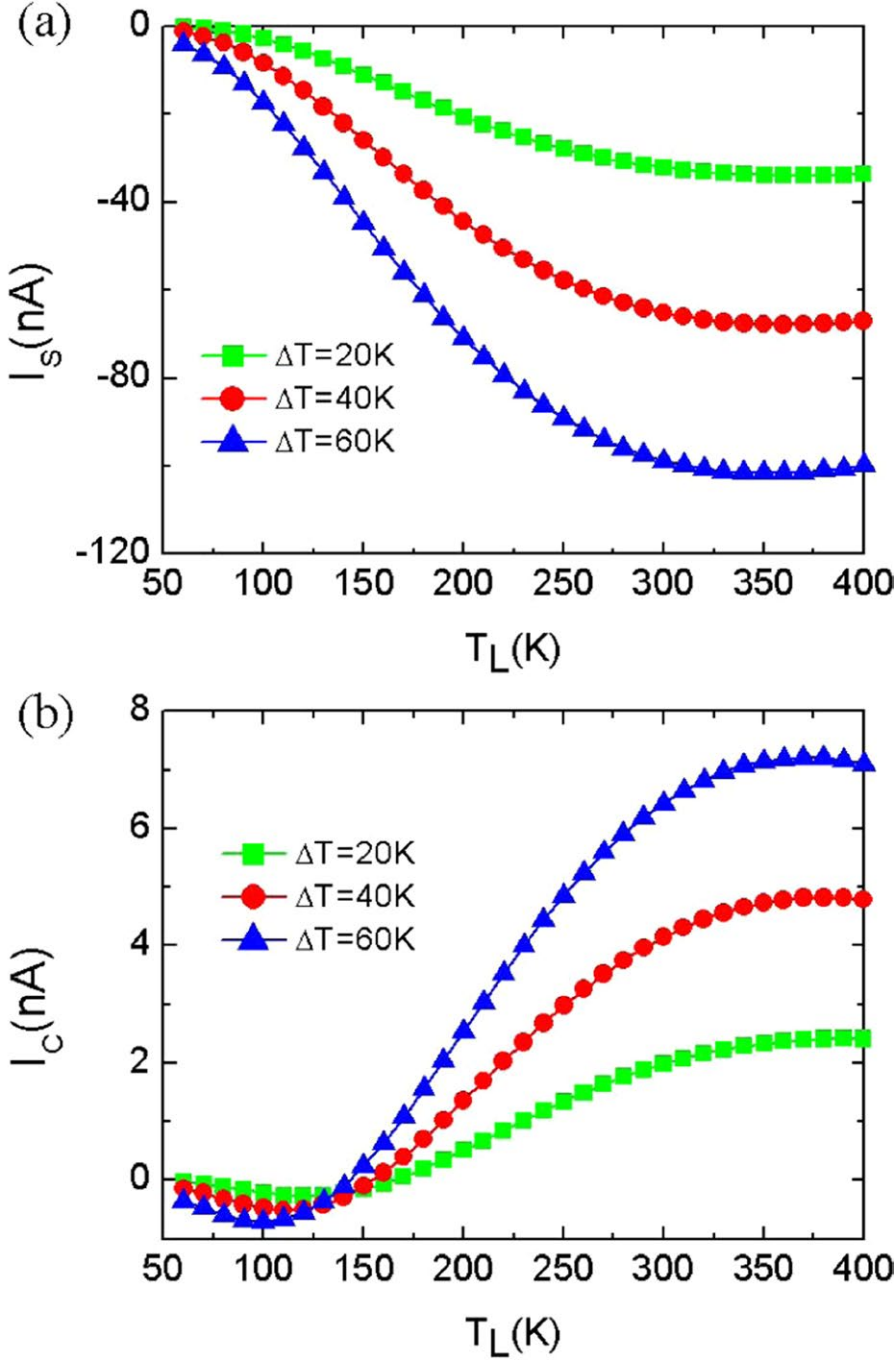
The total charge current and net spin current. The total charge current *I*_*C*_ (*I*_*up*_ + *I*_*dn*_) (**a**) and the net spin current *I*_*S*_ (*I*_*up*_ − *I*_*dn*_) (**b**) of Fe-C_6_ device as a function of *T*_*L*_ for P spin configuration.

We now discuss the charge currents *I*_*C*_ (*I*_*up*_ + *I*_*dn*_) and spin currents *I*_*S*_ (*I*_*up*_ − *I*_*dn*_) as a function of *T*_*L*_ to describe the SSE. As can be seen from Fig. 3, *I*_*S*_ is about 10 times larger than *I*_*C*_ for each of *∆T*. When *∆T* ranges from 20 K to 60 K, *I*_*S*_ increases. It is noteworthy that *I*_*C*_ shows unusual behavior. We take *∆T* = 60 K as an example. As *T*_*L*_ increases, *I*_*C*_ declines to negative value, and reaches to its minimum −0.7 nA at *T*_*L*_ = 100 K, after that, *I*_*C*_ begins to increase to almost zero (about −0.09 nA) at 140 K. Whereafter, *I*_*C*_ continues to increase with increasing *T*_*L*_, and finally reaches the maximum value 7.21 nA at *T*_*L*_ = 370 K, then decreases again. The novel property of *I*_*C*_ is due to the competition between *I*_*up*_ and *I*_*dn*_, videlicet, the result of the competition between the spin up hole carriers and the spin down electron carriers in the heat transport. The nonlinear response of *I*_*C*_ is very necessary for the design of thermal transistors. At *ΔT* = 60 K, *T*_*L*_ = 140 K, the value of *I*_*C*_ is −0.09 nA, but *I*_S_ is −8.87 nA. Although at *T*_*L*_ = 150 K, *I*_C_ is very small (0.24 nA), but the absolute value of *I*_*S*_ is also large (*I*_*S*_ = −44.62 nA). The above results indicate that the net spin current is produced at *ΔT* = 60 K within the *T*_*L*_ region (140 K, 150 K), and the total charge current is well suppressed. Thus the perfect SSE occurs.

Here, the electrode leads are the same material, and the left electrode is colder than the right electrode. When the source and drain have different temperature, the behavior of carrier is determined not only by transmission function, but also by the Fermi distribution difference ${f}_{L}(E,{T}_{L})-{f}_{R}(E,{T}_{R})$ which is only related to the temperature *T*_*L*/*R*_ of source/drain. If the transmission functions of spin-up and spin-down channels are strong symmetric around Fermi level, the electron current (*I*_*e*_) and hole current (*I*_*h*_) will be cancelled each other and the device will not have total charge current, only net spin current is produced. In Fe-C_6_ device, the spin down carriers (electrons) flow from drain to source, producing the positive current, while the current generated by the spin-up carriers (holes) flowing from source to drain is negative, then there is a nonzero spin current in the P configuration.

**Figure 4:**
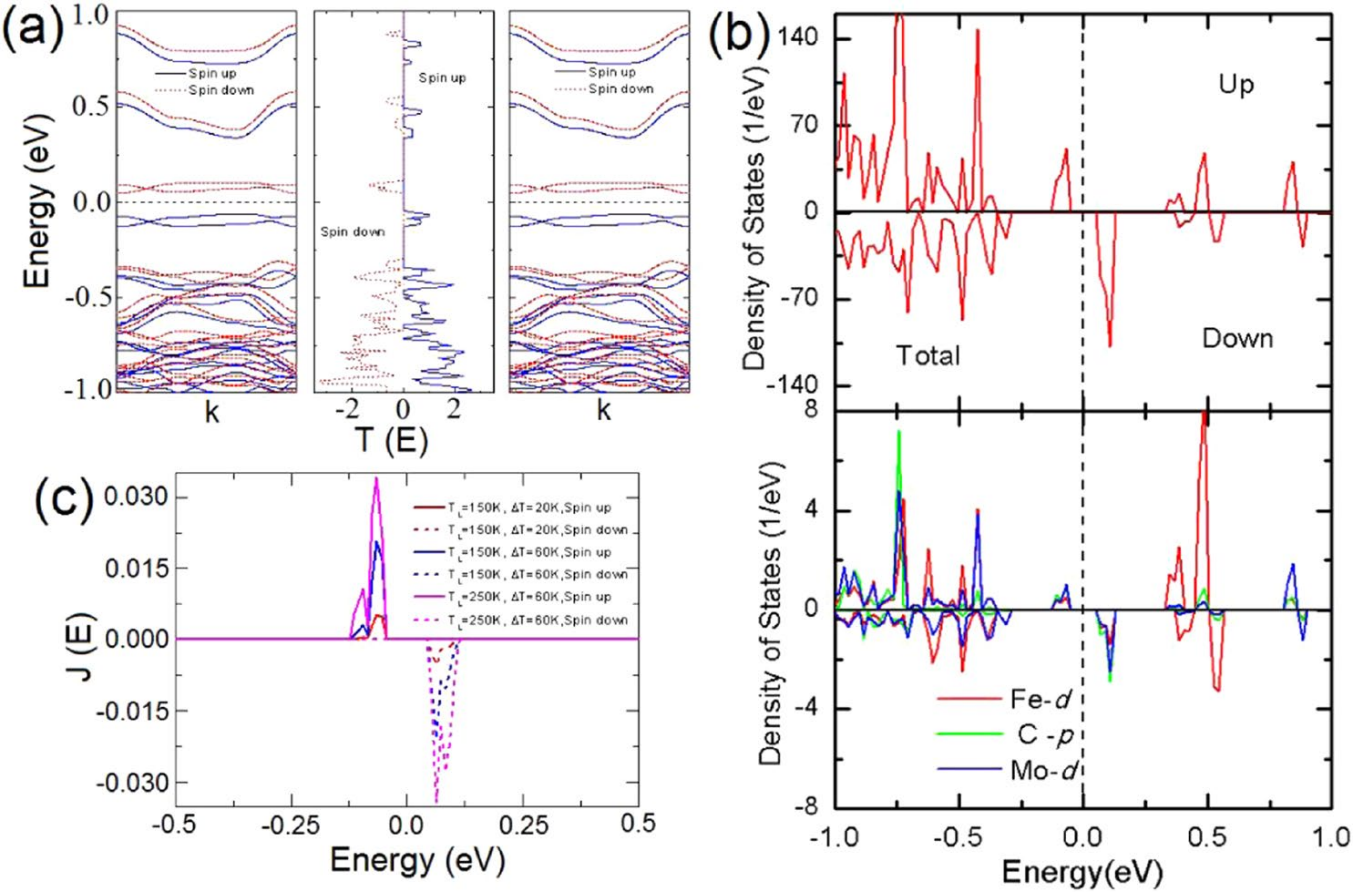
The spin-resolved electronic structures and the current spectra. (**a**) Band structures of electrode (the left panel is the same as the right one) and the spin-polarized transmission spectrum for P (middle panel) spin configuration. (**b**) The electronic structures for Fe-C_6_ device and atomic orbital. (**c**) Spin-polarized current spectra for various *T*_*L*_ and *∆T*.

To elucidate the physical mechanism for SSE, we analyze the band structure of Fe-C_6_ doped monolayer MoS_2_ system and the spin dependent transmission function of Fe-C_6_ device, as shown in Fig. 4(a). The mechanism of the current generated from temperature difference credits to the electron band structure. The left panel of Fig. 4(a) manifests that the spin-up and spin-down bands are split and have a good symmetry near the Fermi level, thus the Fe-C_6_ doped system is a spin semiconductor. The transmission function (the middle panel of Fig. 4(a)) shows that the spin-up and spin-down transport channels are both opened near the Fermi level for P spin configuration, due to the match of the bands of the left and right electrode. Accordingly, transmission peaks near the Fermi level present in the energy range (−0.124 eV, −0.045 eV) and (0.055 eV, 0.114 eV) for spin-up and spin-down channels, respectively. Because they are very close to the Fermi level, the temperature threshold is small. These two peaks break the electron-hole symmetry in the transmission function, resulting in the nonzero net thermal spin currents. Additionally, the transmission peaks of both channels are almost symmetrical about the Fermi level, thereby, Fe-C_6_ device produces a perfect SSE. The spin splitting of the density of states (DOS) in Fig. 4(b) verifies the spin-polarized transport. Furthermore, the spin up and down DOS are nearly symmetric about the Fermi level, therefore, there should be SSE. The lower panel of Fig. 4(b) reveals that it is the *d*-states of Fe, *p*-states of the nearest neighbor C, and *d*-states of the second-nearest neighbor Mo contributing to the thermal transport properties.

Now, we analyze the current spectra ($J(E)=T(E)({f}_{L}(E,{T}_{L})-{f}_{R}(E,{T}_{R}))$) (Fig. 4(c)), which reflects the value of current. The spin-up and spin-down current spectra both augment with the increasing of *T*_*L*_ or *∆T*, and they are almost equal in area, resulting in numerically approximate equal of spin up and spin down currents with opposite signs. These phenomena further confirm the emergence of SSE.

**Figure 5:**
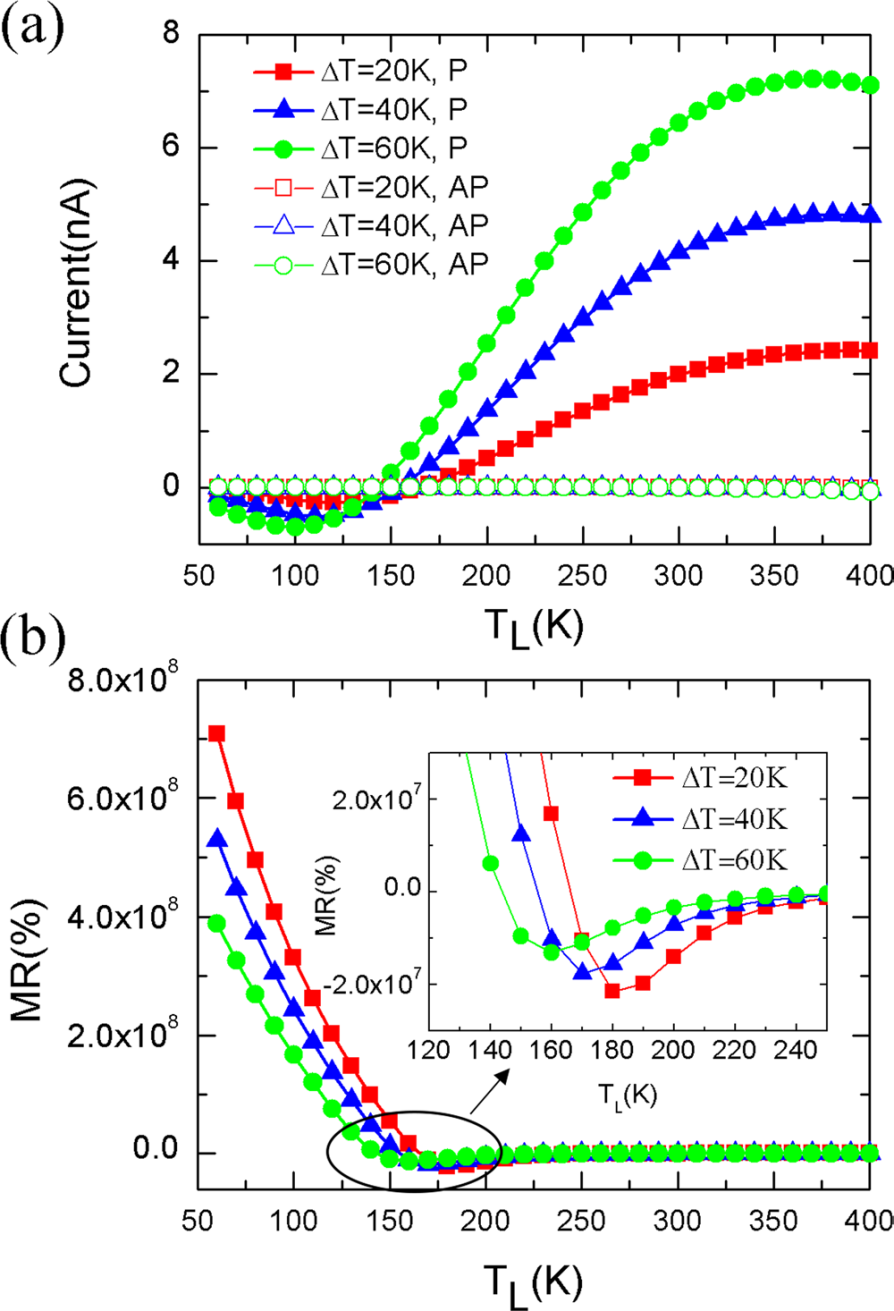
The total charge current and the thermal magnetoresistance. (**a**) The total charge current as a function of *T*_*L*_ for P and AP spin configurations with *∆T* = 20, 40 and 60 K. (**b**) The thermal magnetoresistance as a function of *T*_*L*_. For clarity, the inset shows the magnetoresistance at the flex point with a smaller scale.

When the device is transformed from P spin configuration to AP spin configuration, the CMR effect of the device appears, $MR( \% )=({I}_{C}^{P}-{I}_{C}^{AP})/{I}_{C}^{AP}\times 100$. Figure 5(a) display that the total charge current of AP spin configuration (${I}_{C}^{AP}$) is smaller than that of P spin configuration (${I}_{C}^{P}$), owing to the transport channel of P spin configuration is closer to the Fermi level than that of AP spin configuration. ${I}_{C}^{P}$ changes from negative value to positive value with increasing *T*_*L*_, but its absolute value at most *T*_*L*_ points is much larger than ${I}_{C}^{AP}$. Figure 5(b) denotes that the CMR reaches 10^7^% (or −10^7^%) in wide range of *T*_*L*_ and is accompanied by the change of symbol, which has special significance and will be widely used in the logic devices. The reason for above phenomenon is that ${I}_{C}^{P}$ varies from negative to positive sign with increasing *T*_*L*_ (see Fig. 5(a)) and is much larger than ${I}_{C}^{AP}$ which is negative and almost zero, thus positive and negative magnetoresistances emerge as indicated by the insert of Fig. 5(b).

The thermal magneto-resistance behavior of Fe-C_6_ doped MoS_2_ is very similar to that of Fe-doped MoS_2_^37^, but the mechanism is disparate. The negative magnetoresistance of Fe-C_6_ doped MoS_2_ system is the result of competition between spin up holes and spin down electrons of P spin configuration. The negative magnetoresistance of Fe-doped MoS_2_ system is caused by the sign change of total charge current of AP spin configuration^37^. The CMR shows a strong regularity within several *∆T* and its value gradually moves close to zero until finally reaches to −10^4^% in Fe-C_6_ device. For convenience, we also call the negative magnetoresistance phenomenon ‘Zigzag’. The behavior of ‘Zigzag’ shifts to low *T*_*L*_ with increasing *∆T*. Negative magnetoresistance appears in the lower *T*_*L*_ with a higher *∆T*, which is in agreement with that of Fe-doped MoS_2_ system. However, the maximum negative magneto-resistance value begins to increase when *∆T* decreases. Namely, the magnetoresistance of *∆T* = 60 K is less than that of *∆T* = 40 K, which is also less than that of *∆T* = 20 K. Based on temperature difference and magnetic field, one can control carriers transport to change the magnetoresistance of Fe-C_6_ doped monolayer MoS_2_ two-probe system, the thermal CMR is higher than 10^7^% and accompanied with the conversion between negative value and positive value. Our findings may provide a good reference to experiment.

We also consider the size effect on the thermally transport. For P spin configuration, the phenomenon of the thermal transport is almost not affected by the length of the scattering region, and the perfect SSE arises yet. The tendency of thermally induced CMR is not changed when *T*_*L*_ increases, but in many of the *T*_*L*_ points, its values augment nearly two orders of magnitude when the length of the scattering region is enlarged from two doped units to four doped units, reaching 10^10^% because of the decrease of *I* ^*AP*^. Meanwhile, the ‘zigzag’ phenomenon of CMR still exists. It can be concluded that the thermal transport properties are enhanced by aggrandizing the length of scattering region.

## Conclusions

In conclusion, a new configuration of spin caloritronic device based on Fe-C_6_ cluster doped monolayer MoS_2_ was presented, and its thermal transport properties had been investigated by NEGF-DFT approach. Spin-polarized currents can be produced only by applying temperature gradient between the left and the right electrodes. The SSE is observed in the thermal electron current. An intriguing thermally induced CMR without gate regulating occurs in the device, which has different sign in distinct *T*_*L*_ region while *∆T* is fixed. The peculiar behavior of CMR and the nonlinear response regime of *I*_c_ arise from the competition between spin-up electrons and spin-down holes of the P spin configuration. Additionally, we find that the thermal transport properties of enlarged scattering region are enhanced. The results manifest that the MoS_2_-based materials have potential application in spin caloritronics and spintronics.
